# The effect of H1N1 vaccination on serum miRNA expression in children: A tale of caution for microRNA microarray studies

**DOI:** 10.1371/journal.pone.0221143

**Published:** 2019-08-20

**Authors:** Ruth Elizabeth Drury, Andrew John Pollard, Daniel O’Connor

**Affiliations:** 1 Oxford Vaccine Group, Department of Paediatrics, University of Oxford, Oxford, Oxfordshire, United Kingdom; 2 NIHR Oxford Biomedical Research Centre, Oxford, Oxfordshire, United Kingdom; Saint Louis University, UNITED STATES

## Abstract

**Background:**

MicroRNAs (miRNAs) are a class of small regulatory RNAs around 21–25 nucleotides in length which govern many aspects of immunity including the host innate and adaptive responses to infection. RT-qPCR studies of select microRNAs show that vaccination alters the expression circulating microRNAs but the effect of vaccination on the global microRNA population (i.e. micronome) has never been studied.

**Aim:**

To describe vaccine associated changes in the expression of microRNAs 21 days after vaccination in children receiving a pandemic influenza (H1N1) vaccination.

**Method:**

Serum samples were obtained from children aged 6 months to 12 years enrolled in an open label randomised control trial of two pandemic influenza (H1N1) vaccines, in which participants received either ASO3B adjuvanted split virion or a whole virion non-adjuvanted vaccine. MicroRNA expression was profiled in a discovery cohort of participants prior to, and 21 days after vaccination using an Agilent microarray platform. Findings were followed up by RT-qPCR in the original discovery cohort and then in a validation cohort of participants taken from the same study.

**Results:**

44 samples from 22 children were assayed in a discovery cohort. The microarray results revealed 19 microRNAs were differentially expressed after vaccination after adjustment for multiple testing. The microarray detected ubiquitous expression of several microRNAs which could not be validated by RT-qPCR, many of which have little evidence of existence in publicly available RNA sequencing data. Real time PCR (RT-qPCR) confirmed downregulation of miR-142-3p in the discovery cohort. These findings were not replicated in the subsequent validation cohort (n = 22).

**Conclusion:**

This study is the first study to profile microRNA expression after vaccination. An important feature of this study is many of the differentially expressed microRNAs could not be detected and validated by RT-qPCR. This study highlights the care that should be taken when interpreting omics biomarker discovery, highlighting the need for supplementary methods to validate microRNA microarray findings, and emphasises the importance of validation cohorts. Data from similar studies which do not meet these requirements should be interpreted with caution.

## Introduction

A greater understanding the immune response can be derived by identifying changes in gene expression after vaccination[1–3]. In order to fully understand immune responses, the regulatory mechanisms behind those changes in gene expression must be investigated. These regulatory mechanisms include the action of non-coding RNAs.

MicroRNAs (miRNAs) are a class of small regulatory non-coding RNAs, around 21–25 nucleotides long, which post-transcriptionally regulate the expression of protein coding genes. They do this by binding to complementary sequences on the 3’ untranslated region of mRNA molecules, thereby inhibiting mRNA translation[4]. 2675 human miRNAs are listed in the microRNA registry (miRbase), and are estimated to collectively regulate around 60% of protein-coding genes [5,6]. MiRNAs play a key role in many cell processes, including those involved in host response to infection[7,8]. MicroRNAs target proteins involved in leukocyte development and differentiation, innate and adaptive immune pathways (e.g. leukocyte activation, cytokine release, antibody affinity maturation) and they may even target viral genomes and transcripts[9–18].

Although miRNAs are primarily intracellular molecules, miRNAs are detected in most body fluids: these extracellular miRNAs can exist in extracellular vesicles (exosomes, microvesicles and apoptotic bodies) or can be associated with argonaut protein and high-density lipoprotein, and, unlike mRNA, show remarkable stability in stored clinical samples[19–24]. The biological function of extracellular miRNAs is debated: but an increasingly accepted theory is that they are secreted as intercellular communicators of gene regulation[25]. Over 56 studies show an association between infectious disease and circulating miRNA expression suggesting that induction of an immune response may alter miRNA release. Given infection influences circulating miRNA expression, then so too may vaccination[26]. The stability and ease of sample acquisition in clinic makes extracellular miRNAs attractive biomarkers to provide new correlates of protection or vaccine reactogenicity. Interrogating the functional effects of extracellular miRNAs on immune response could provide new insights into vaccine-mediated immunity which can be exploited to improve vaccine design. RT-qPCR studies of candidate miRNAs have shown vaccination alters serum miRNAs and this is associated with vaccine response [27–30].

To our knowledge, however, no published studies have used a whole miRNA profiling platform to investigate the effect of vaccination on global serum/plasma miRNA expression. The aim of this study was to use a whole miRNA profiling technique to investigate the effect of two pandemic influenza (H1N1) vaccinations (ASO3B adjuvanted split virion and a whole virion non-adjuvanted vaccine) on the serum miRNA expression of children 3 weeks after the completion of their vaccination course compared with baseline.

## Materials and methods

Participant samples were obtained from an open label, randomised, parallel group, UK multicentre study assessing the safety and immunogenicity of two novel pandemic influenza (H1N1) vaccines: ASO3B adjuvanted split virion (GlaxoSmithKline Vaccines, Rixensart, Belgium) or a whole virion non-adjuvanted vaccine (Baxter Vaccines, Vienna) (clinical trial registration number: NCT00980850[31])clinical trial. Between 26 September and 11 December 2009, during the second wave of the influenza A (H1N1) pandemic in the UK, children aged 6 months to 12 years were enrolled in the trial. Children whose parents provided informed consent and were able to comply with study procedures were included. Key exclusion criteria included prior receipt of a H1N1 vaccination, confirmed pandemic H1N1 influenza infection, severe immunocompromise, or receipt of immunosuppressive treatment. Block randomisation was undertaken stratified by age.

**Fig 1:**
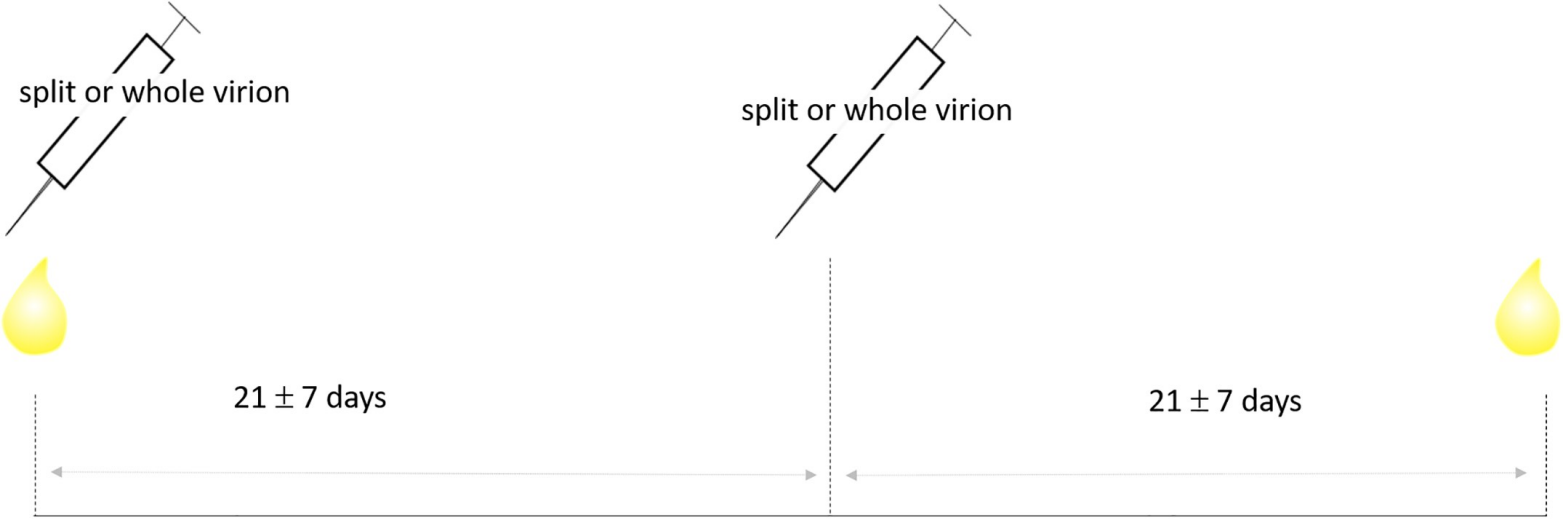
Study design. Participants were randomised to receive a 2-dose course (21 ± 7 days between doses) of an ASO3B adjuvanted split virion or a whole virion non-adjuvanted vaccine. Serum samples used for microarray analysis and RT-qPCR were collected immediately before vaccination and 21 days (minus 7 days to plus 14 days) after second vaccination.

Vaccines were administered by intramuscular injection at enrolment and at day 21 (plus or minus 7 days). Serum samples were collected immediately before vaccination and 21 days (minus 7 days to plus 14 days) after second vaccination (Fig 1). Antibody responses were measured by microneutralisation and haemagglutination inhibition using standard methods at the Centre for Infections, Health Protection Agency[31–33].Seroconversion was defined as a fourfold rise to a titre of ≥1:40 from baseline 3 weeks post 2^nd^ vaccine dose. Full details of the trial can be found in Waddington et al[31]. The trial was approved by the Oxfordshire Research Ethics Committee A (No 09/H0604/107), the UK Medicines and Healthcare Products Regulatory Agency (EUDRACT 2009-014719-11), and local NHS organisations by an expedited process.

### Sample cohorts

MiRNA profiling was conducted on pre-vaccine and three weeks post 2^nd^ vaccine plasma samples from a random selection of 22 participants. This comprised the discovery cohort. The validation cohort comprised of a second random selection (stratified by type of vaccine received) of 22 participants. A power calculation was conducted to inform the sample size of the validation cohort. pwr.t.test in the “pwr” R package was used to perform a power calculation for a one sample, one tailed, t-test using the mean and standard deviation of the log_2_ fold-change derived from the RT-PCR results in the discovery cohort [1]. A one tailed t-test was used based on the direction of the statistically significant log_2_ fold-change seen in the discovery cohort. We chose a sample size that would be over 90% powered to identify differential expression of the miRNA of interest at a significance level of 0.05. For full details of the power analysis and the parameters used see S1 Results.

### Serum miRNA extraction

Total RNA was extracted from 200 microlitres of previously frozen sera using the Total RNA Extraction Kit (Norgen cat# 17200) as specified by the protocol. As expected for RNA extracted from biofluids, RNA concentration was below detectable limit when measured by spectrophotometry (NanoDrop, Thermoscientific) and flourometer (Qubit, Invitrogen).

### MiRNA expression profiling

MiRNA expression profiling was undertaken in a cohort of 22 randomly selected children using the Agilent miRNA microarray 60K (design 031181, based on miRbase version 16.0) using a one colour experimental design. The Agilent miRNA protocol (Version 2.4 September 2011) was used with the exception that after the labelling with Cy3-pCp molecule samples were dried and ‘Step 2’ was omitted. Samples were hybridised for 40 hours to increase specific signal against background. "[34].

Data analyses were conducted in the statistical language R using Bioconductor packages[35]. MiRNAs detected in less than 50% of samples were filtered out. Where a miRNA was not detected in a sample, its fluorescence value was set to half the value of the minimum detected fluorescence value across all samples for that miRNA. Data were then normalised using variance stabilised normalisation (package vsn version 3.40)[36]. Negative and positive control miRNAs were removed.

Samples were clustered using Ward’s linkage method which is appropriate for expression profiling data[37]. People often have an innate expression profile which is unique to them–because of this, variation between people is can be greater than vaccine induced variation within a person. As a result, paired expression profiles tend to cluster together. The effect of pairing on cluster analyses was removed using a batch correction function in R called RemoveBatchEffect from the package limma, with participant identification number as a batch effect[37]. This function fits a linear model to the data, then removes the component due to the batch effects (in this case participant number).

Differential miRNA expression was tested using multivariable linear regression using participant ID number (to account for pairing in the data) and vaccine status (“pre-vaccine”, “post-vaccine”) as factors in the model. Sex, age and vaccine type were not significantly associated with miRNA expression so were not included as variables in the final model. The resulting p-values for each miRNA were adjusted for multiple testing using the Benjamini-Hochberg false discovery rate (FDR).

### Selection of appropriate endogenous control for RT-qPCR data normalisation

RT-qPCR of miRNA in serum is challenging as there is no scientific consensus on the most appropriate miRNA to be used as an endogenous control. Commonly used endogenous controls like miR-16 and the small nuclear RNA U6 differ from person to person and can be affected by sampling factors like haemolysis[38,39]. Spike-in controls (synthetic/non-human small RNAs added to samples prior to miRNA extraction) do not allow for inter-individual differences in global miRNA expression[40]. Candidate endogenous controls were therefore selected from the microarray data. Candidate endogenous control miRNAs had to be present in 100% of samples, have a minimal average fold-change in expression pre and post vaccine and have minimal variance in expression between paired pre and post-vaccination samples. To facilitate identification of potential reference miRNAs, miRNAs present in 100% of samples were ranked in terms of their average log fold-change, and in terms of the standard deviation of the fold-changes. These ranks were then multiplied together to give a “composite” rank. MiRNAs were then ranked by their composite rank and miRNAs with the lowest composite rank were selected as potential controls.

Reanalysis of log2 transformed unnormalized microarray miRNA expression levels normalised using the expression levels each of each candidate reference miRNA, reproduced significant differential expression of the miRNAs of interest, confirming their suitability as a candidate endogenous control. Ideally three endogenous reference genes should be used which have been shown to be stably expressed across conditions[41]. This was our intention but only one out of six assays for our candidate reference miRNAs could be optimised. This optimised assay was for miR-29c.

### Quantitative reverse transcription PCR (RT-qPCR)

RT-qPCR was used to confirm microarray results. In the first instance, miRNA specific assays using miRNA Locked Nucleic Acid (LNA) primer sets (Exiqon) were used to qualitatively measure miRNA expression (cat# 339306, see S1 Table for assay IDs). LNA primers were chosen because they have a higher maximum annealing temperature leading to higher specificity (can discriminate highly homologous miRNAs from the same family) compared with standard primers, and they have been shown to be highly sensitive (very desirable for RNA poor biofluids)[42–45]. Lyophilised primers were resuspended in 220 μl of nuclease free water. Two microlitres of total RNA was universally reversed transcribed using the Universal cDNA Synthesis Kit II (Exiqon cat# 203301) according to the manufacturer instructions. A synthetic oligonucleotide, UniSP6 (Exiqon cat# 203301), was spiked in to each reverse transcription reaction to identify any samples which failed/were outliers in the reverse transcription step. cDNA was then subject to qPCR. Each PCR well contained 0.1μl cDNA, 1μl primer, 5μl 2X IQ SYBR Green supermix (Bio-Rad, cat# 170–8882), 0.2μl ROX reference Dye (Thermofisher, cat # 12223012), 3.7μl of RNAase free water to give a final reaction volume of 10μl and amplified using the following conditions: 95°C for 10 min, followed by 40 amplification cycles at 95°C for 10s and 60°C for 1 min. A post PCR melt curve was performed on each sample for each assay. Only assays with primers efficiencies between 95–105% were taken forward.

TaqMan Advanced miRNA assays (cat# A25576) were used as second line for several miRNA where LNA primers failed to detect their targets (see S1 Table assay IDs). Two microlitres of total RNA was universally reversed transcribed and universally pre-amplified using TaqMan Advanced miRNA cDNA Synthesis Kit (cat# A28007) according to the manufacturer instructions. cDNA was diluted 1:10 with 0.1 X TE buffer then subject to qPCR. Each PCR well contained 2.5μl diluted cDNA, 0.5μl primer, 5μl 2X TaqMan Advanced Master Mix (cat# 4444556) and 2μl RNAase-free water to give a final reaction volume of 10μl and amplified using the following conditions: 95°C for 20 seconds, followed by 40 amplification cycles at 95°C for 1 second and 60°C for 20 seconds.

In all cases, qPCR was conducted using the StepOnePlus Real-Time PCR System (Thermofisher, cat # 4376600). Each assay was conducted in triplicate and an arithmetic mean cycle threshold values (Ct values) calculated. Negative controls without cDNA were included on each PCR plate. Assays were only taken forward if their products met the following criteria for acceptable amplification: amplification curves that were within the limits of detection (Cts < 35 and 5 Cts lower than background Ct values in non-template control wells), had a single Tm peak in melt curve analysis (in the case of LNA primers) and showed evidence of amplification (amplification curve seen when normalised florescence, Rn, is plotted on a linear scale). Positive control RT-qPCR assays for the spiked in UniSp6 oligonucleotide were run for each sample.

The mean Ct for each sample assay was normalised to an endogenous reference miRNA (see below) which was stably expressed between pre- and post-vaccination samples as determined from the microarray data (see Results). Relative expression between paired pre- and post-vaccination samples was calculated using the 2^-ΔΔCt^ method.

To test for differential expression of a miRNA pre- and post-vaccination using RT-qPCR data a one sample Student’s t-test was used to test the null hypothesis that the log_2_ fold-change between pre and post vaccine samples were 0 (equivalent to a relative fold change of 1). In the case of the discovery cohort a two tailed t-test was used, in the case of the validation cohort a one tailed t-test was used based on the direction of change seen in the validation cohort. Average log_2_ fold-change were exponentiated to 2 to give average fold-change.

## Results

### Participants

MiRNA profiling was conducted on samples from a random selection of 22 participants. This comprised the discovery cohort. The validation cohort comprised of a second random selection of 22 participants who were stratified by type of vaccine received. Demographics are listed in Table 1. A further breakdown of each cohort by gender and vaccine type can be found in supplementary materials (S2–S5 Tables).

**Table 1:** Participant demographics and vaccine group.

Cohort	N	Vaccine % AS03B adjuvanted split virion vaccine	Sex % Female	Age (years) Median (IQR)
Discovery cohort	22	41	55	5.69 (3.25–9.30)
Validation cohort	22	50	50	3.77 (2.61–8.96)

Although the validation cohort was slightly younger, all cohorts were similar in their demographics

### Microarray analysis showed that vaccination changed global miRNA expression

**Fig 2:**
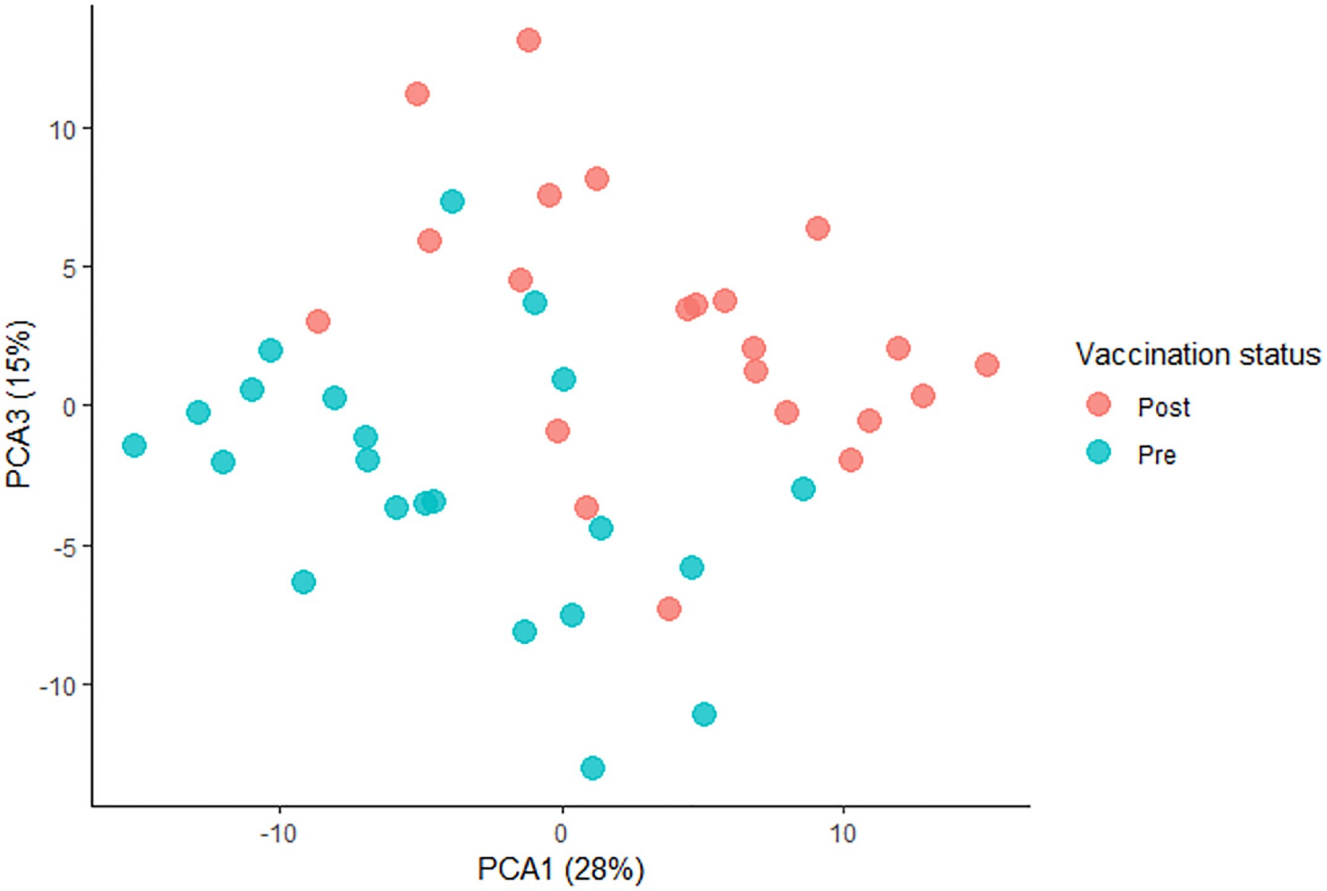
Principal component analysis based on each sample’s microRNA expression. All miRNAs passing QC were included in the principle component analysis, after adjusting for pairing in the data*. PCA 1 and PCA 3 are shown. Pre-vaccine samples are coloured blue, post vaccine samples are coloured red. There is some overlap between the pre- and post- vaccine sample clusters, nevertheless, there is a tendency for pre-vaccine sample to cluster to lower left, and pre-vaccine samples to cluster to upper right. This suggests that some but not all variation in global microRNA expression is accounted for by vaccine status. Clustering is still seen even if there is no adjustment for pairing in the data (S1 Fig). *Samples from the same individual have a tendency to cluster together when inter-individual variation is greater than intra-individual variation. To overcome the effect of sample pairing in the PCA, participant ID was modelled as a batch effect, and then these “batch-effects” were removed using the RemoveBatchEffect() function in limma.

In total 189 miRNAs passed QC and were included for analysis. PCA analysis showed evidence of clustering of pre- and post-vaccination samples with respect to principle components 1 and 3, indicating that vaccination may affect miRNA expression profile (Fig 2).

### There was significant differential expression of miRNAs after vaccination

**Fig 3:**
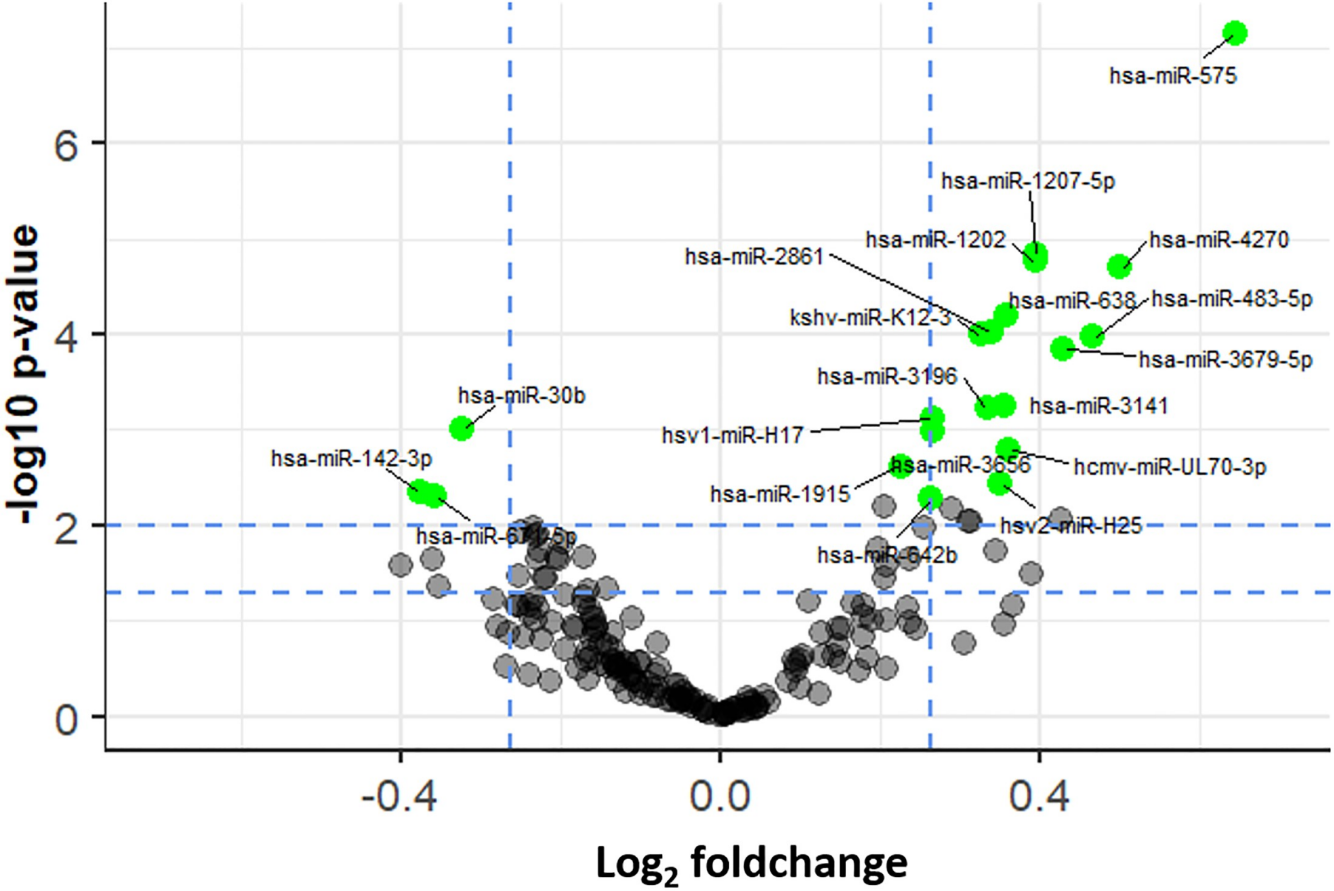
Volcano plot for identification of differentially expressed miRNAs. Green spots represent miRNAs that have an FDR adjusted p-value of <0.05. Blue dashed vertical lines demarcate an up and downregulation fold-change cut-off of 1.2. In total 18 microRNAs were significantly differentially expressed at above this fold-change cut-off.

**Fig 4:**
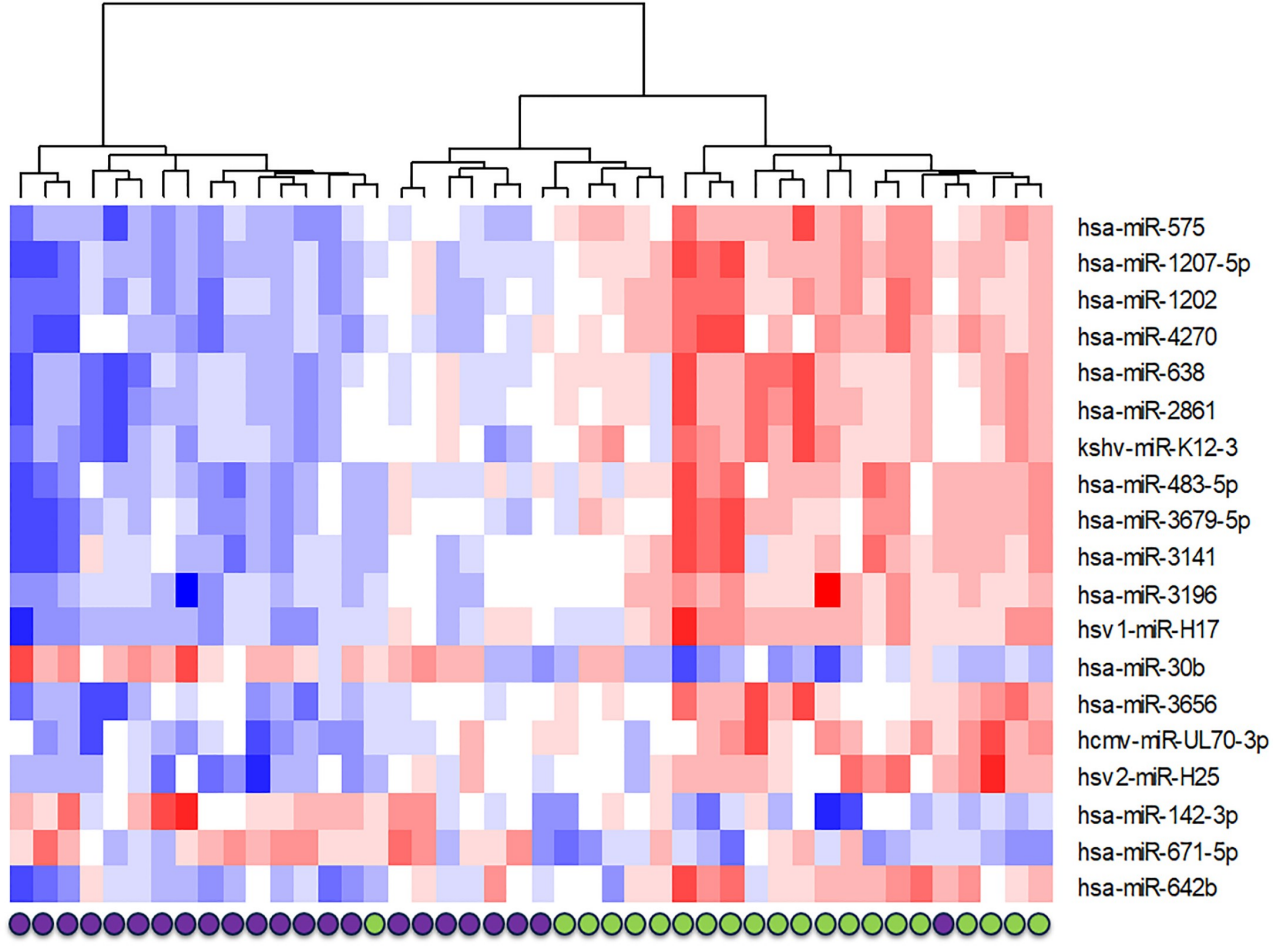
Unsupervised hierarchical clustering based on expression values for significantly differentially expressed miRNAs after the effect of the individual had been removed. Purple dots indicate pre-vaccine samples, green dots indicate post vaccine samples. Based on the expression of microRNAs which were differentially expressed, pre and post vaccine samples could be clearly separated out with the exception of one person’s samples.

Nineteen miRNAs were differentially expressed (16 upregulated, 3 down regulated) pre-vaccination versus three weeks post-vaccination and had a fold-change of a magnitude >1.2 (Fig 3, and Table 2). These miRNAs are listed in Table 1 and include four viral miRNAs, hcmv-miR-UL70-3p (human cytomegalovirus miRNA), hsv2-miR-H25 (a herpes simplex 2 miRNA), kshv-miR-K12-3 (a Kaposi’s sarcoma virus miRNA), and hsv1-miR-H17 (a herpes simplex 1 miRNA). The most upregulated miRNA was miR-575 (fold-change = 1.57 adjusted p-value <0.001) and the most downregulated miRNA was miR-142-3p (fold-change = 0.77, adjusted p-value = 0.004). Unsupervised hierarchical cluster analysis using the 19 differentially expressed miRNAs was able to differentiate pre-vaccination samples from post-vaccination samples in all but one case (Fig 4).

**Table 2:** MiRNAs which were differentially expressed after vaccination.

Direction of fold-change	MiRNA	Mean log_2_ fold-change	Mean fold-change	p-value	Adjusted p-value
Up	**miR-575**	0.65	1.57	<0.001	<0.001
	**miR-4270**	0.50	1.42	<0.001	<0.001
	**miR-483-5p**	0.47	1.38	<0.001	0.003
	**miR-3679-5p**	0.43	1.35	<0.001	0.003
	**miR-1207-5p**	0.40	1.32	<0.001	<0.001
	**miR-1202**	0.40	1.32	<0.001	<0.001
	**hcmv-miR-UL70-3p**	0.36	1.29	0.002	0.021
	**miR-638**	0.36	1.28	<0.001	0.002
	miR-3141	0.35	1.28	0.001	0.010
	**hsv2-miR-H25**	0.35	1.28	0.004	0.042
	miR-2861	0.34	1.27	<0.001	0.003
	miR-3196	0.33	1.26	0.001	0.010
	kshv-miR-K12-3	0.33	1.25	<0.001	0.003
	miR-3656	0.27	1.20	0.001	0.014
	**hsv1-miR-H17**	0.27	1.20	0.001	0.012
	miR-642b	0.26	1.20	0.005	0.049
Down	**miR-30b**	-0.32	0.80	0.001	0.014
	miR-671-5p	-0.36	0.78	0.005	0.048
	**miR-142-3p**	-0.38	0.77	0.004	0.047

(miRNAs that were tested by RT-qPCR are highlighted in bold)

### Selection of candidate reference miRNAs

**Fig 5:**
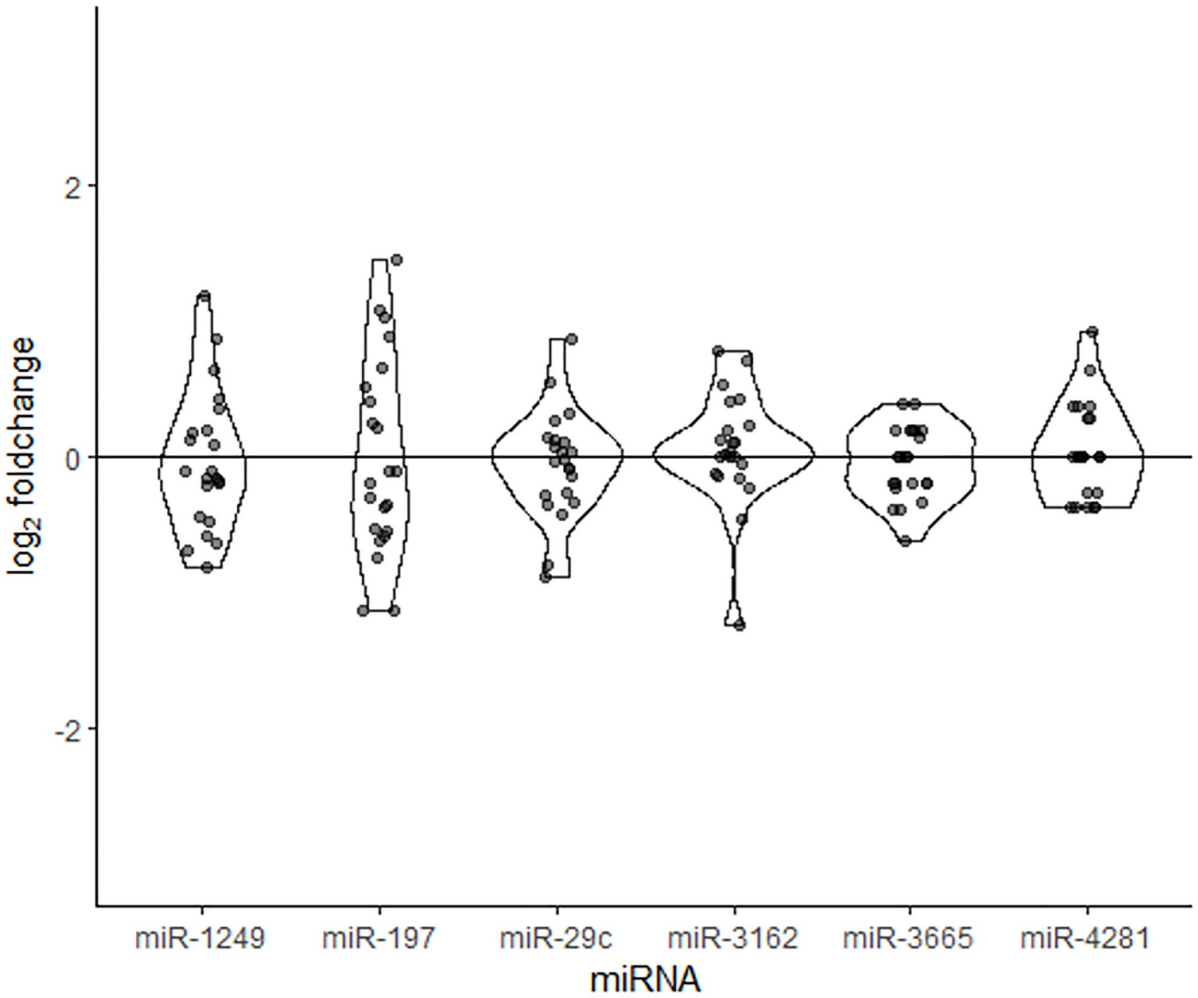
Boxplots of log_2_ fold-changes of candidate reference genes in the miRNA microarray data. Candidate endogenous control miRNAs were selected because they were present in 100% of samples, had a minimal average fold-change in expression pre and post vaccine and had minimal variance in expression between paired pre and post-vaccination samples.

Six miRNAs were selected as candidate reference miRNAs on the basis that they were ubiquitously expressed in all samples and were stably expressed before and after vaccination (Fig 5). MiRNAs selected as possible endogenous references were: miR-3665, miR-3162-5p, miR-29c-3p, miR-1249, miR-197-3p, miR-4281.

### RT-qPCR failed to detect the majority of differentially expressed and candidate endogenous reference miRNAs

Cross platform validation using RT-qPCR was undertaken to confirm the findings of the microarray data. MiRNAs miR-575, miR-4270, miR-483-5p, miR-3679-5p, miR-1207-5p, miR-1202 were selected for initial validation as they had the strongest and most significant fold-changes. None of these miRNAs could be reliably detected by RT-qPCR. The assays for some of these miRNAs had not been wet-lab validated by the manufacturer. Differentially expressed miRNAs which had pre-validated primers were therefore pursued instead: miR-638, hsv1-miR-H17, miR-30b-5p, hcmv-miR-UL70-3p, hsv2-miR-H25 miR-142-3p and miR-671-5p. Of these miRNAs only miR-30b-5p and miR-142-3p met the criteria for acceptable amplification detectable in serum. In a final attempt to detect the remaining differentially expressed miRNAs, TaqMan advanced miRNA assays were used to assay for miR-575, miR-483-5p, miR-3679-5p and miR-638. Of these only miR-483-5p was detectable. miR-29c and miR-197-3p were the only candidate reference miRNAs that were detectable using RT-qPCR, but only miR-29c fully met the criteria for an acceptable assay because Ct values for miR-197-3p were within 4 Cts of non-template controls and primer efficiency was <95%.

### RT-qPCR analysis confirmed downregulation of miR-142-3p

**Fig 6:**
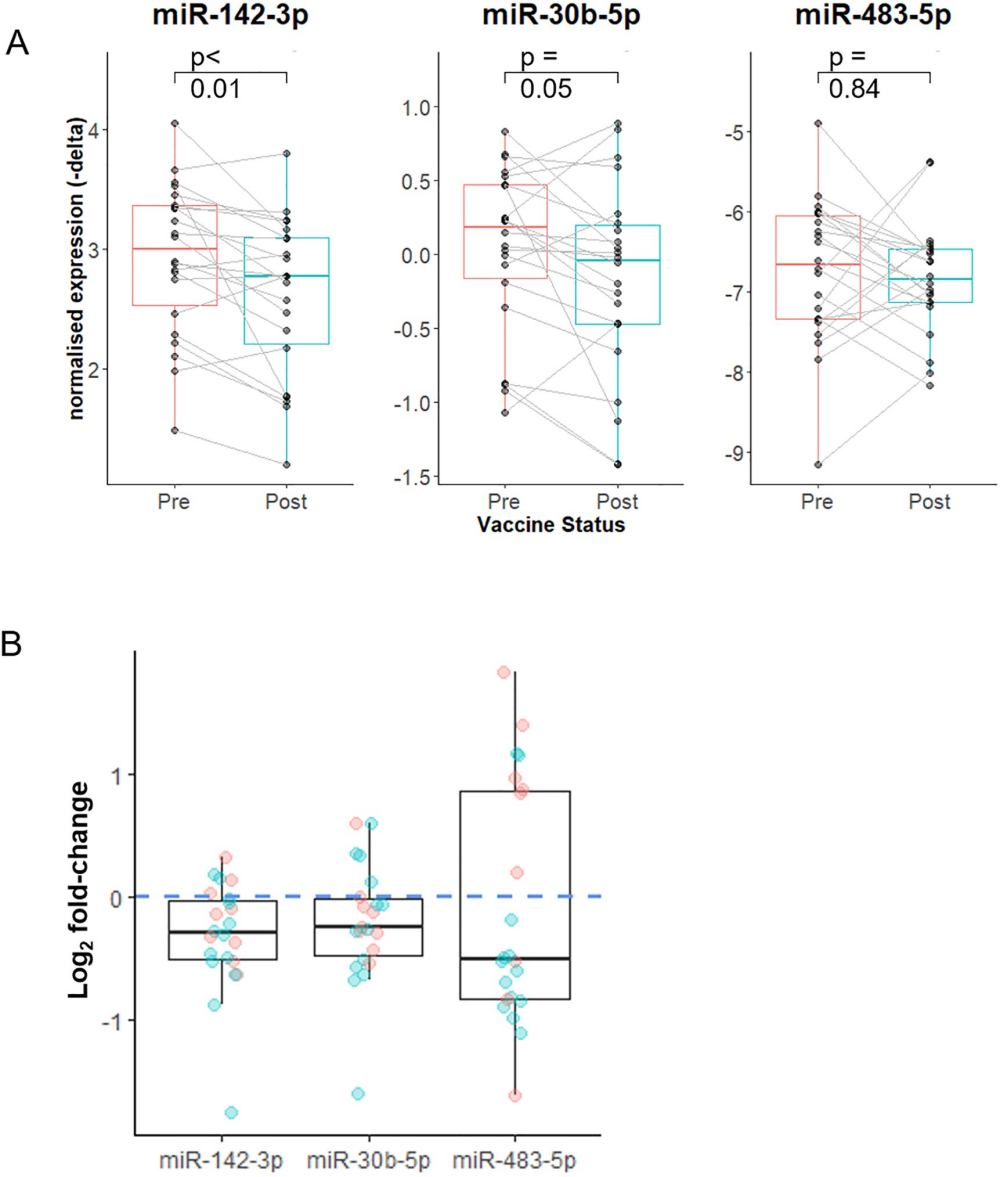
Expression of miR-142-3p, miR-30b and miR-483-5p after vaccination in the discovery cohort measured by RT-qPCR. (A) Expression of miR-30b, miR-142 and miR-483-5p relative to miR-29c pre and post vaccine. Paired samples joined by lines. (B) Log_2_ fold-change in miRNA expression pre versus post vaccination. There was significant downregulation of miR-142-3p. A log_2_ fold-change of 0 (i.e. no change in expression) is highlighted the horizontal blue dashed line. Red colour indicates female participant, blue indicates male participant. Sex did not have a significant effect on magnitude of fold-change.

RT-qPCR on samples from the 22 participants’ in the discovery cohort confirmed significant downregulation miR-142-3p (mean fold-change = 0.805, log_2_ fold-change = -0.312, sd = 0.44, p = 0.003) (Fig 6) validating the microarray results for this miRNA. Downregulation of miR-30b was of borderline significance (mean fold-change = 0.86, mean log_2_ fold-change = -0.21, sd = 0.47, p = 0.05). miR-483-5p was not downregulated (mean fold-change = 0.93, mean log_2_ fold-change = -0.1, sd = 0.97, p = 0.64). There was no evidence that the type of vaccine received correlated with fold-change in miR-142-3p expression in the discovery cohort. The effect size (mean log_2_ fold-change/ standard deviation of the log_2_ fold-change) in the split virion group was -0.73 which is very similar to the effect size of the whole-virus vaccine -0.70. Raw expression (i.e. non-normalised expression) of miR-29c was more stable than raw expression of miR-142-3p and miR-30b supporting the use of miR-29c as an endogenous control for this cohort (see supplementary materials, S4 Table).

### Downregulation of miR-142-3p was not replicated in an independent validation cohort

**Fig 7:**
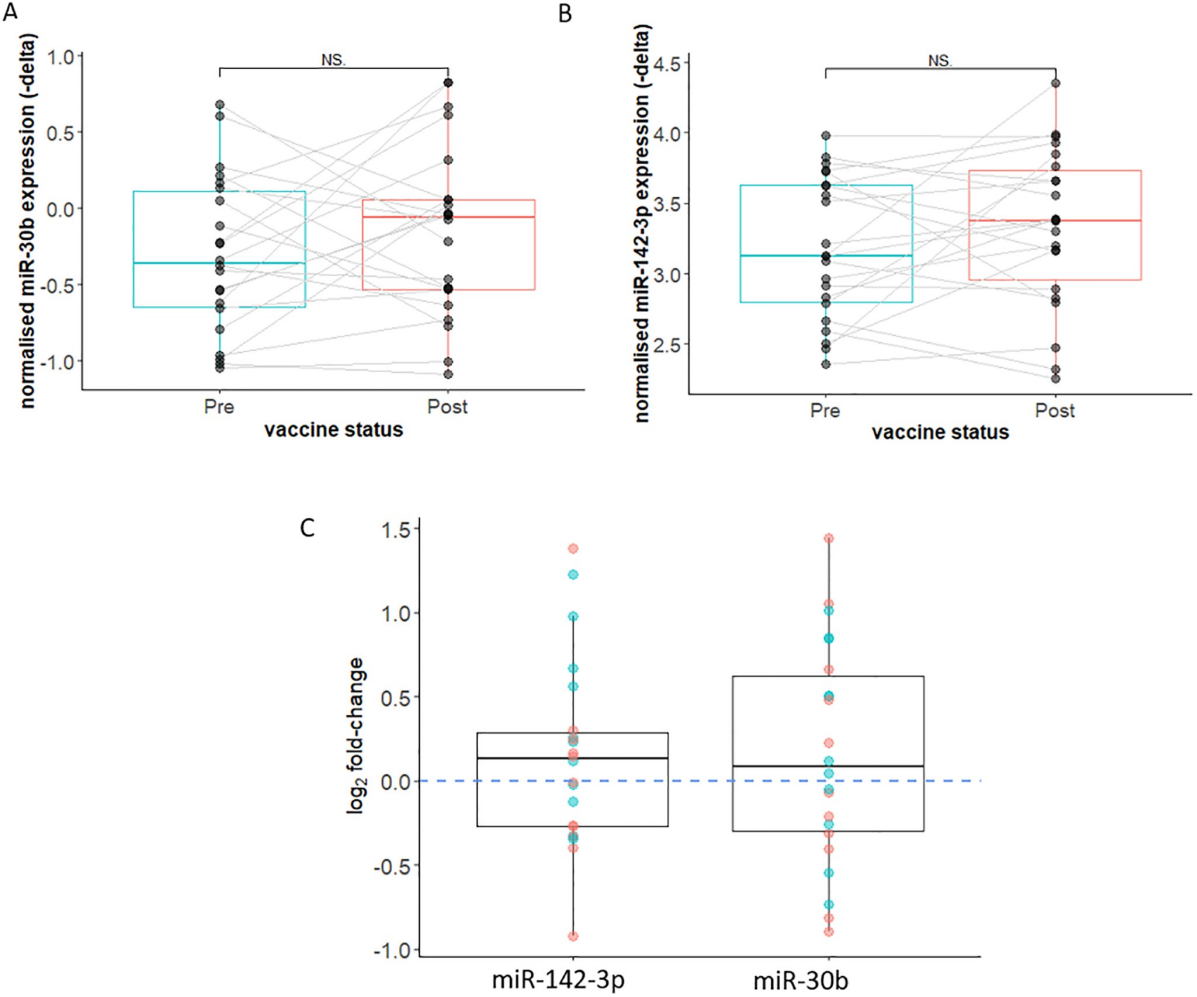
Expression of miR-142-3p and miR-30b after vaccination in the validation cohort as measured by RT-qPCR. **s**(A) Expression of miR-30b relative to miR-29c pre and post vaccine. Paired samples joined by lines, red colour indicates female participant, blue indicates male participant. Sex did not have a significant effect on miRNA expression at baseline, or the fold-change in miRNA expression. (B) Expression of miR-142-3p relative to miR-29c pre and post vaccine. Paired samples joined by lines, red colour indicates female participant, blue indicates male participant. Sex did not have a significant effect on miRNA expression at baseline, or the fold-change in miRNA expression. (C) Log_2_ fold-change in miRNA expression pre versus post vaccination. There was significant downregulation of miR-142-3p. A fold-change of 1 (i.e. not difference) is highlighted the horizontal blue dashed line. Red colour indicates female participant, blue indicates male participant. Sex did not have a significant effect on magnitude of fold-change.

Although miR-30b did not show significant differential expression at a significance value of < 0.05 in the discovery cohort (when measured by RT-qPCR), the result was of borderline significance (p = 0.05) therefore it was also assessed in the independent validation cohort. There was no significant difference in expression of miR-142-3p or miR-30b-5p after vaccination in the independent validation cohort (mean fold-change miR-142-3p = 1.1, log_2_ fold-change = 0.15, sd = 0.55, p = 0.89; mean fold-change miR-30b = 1.11, mean log_2_ fold-change = 0.16, sd = 0.66, p = 0.86), (Fig 7).

### Lack of replication of differential expression of miR-142-3p in an independent validation cohort is not due to selection of miR-29c as an endogenous reference miRNA

Replicating significant downregulation miR-142-3p in the validation cohort is contingent upon the miRNAs being truly differentially expressed and miR-29c being a good endogenous reference in that cohort. A housekeeping miRNA must be consistently non-differentially expressed for it to perform well as a normaliser.

If expression of miR-29c had been more variable between timepoints in the validation cohort (compared with the discovery cohort) then it would have introduced extra technical noise, reducing power for detecting differential expression in the validation cohort. If miR-29c had been downregulated after vaccination in the validation cohort then this would have masked downregulation of miR-142-3p and miR-30b.

**Fig 8:**
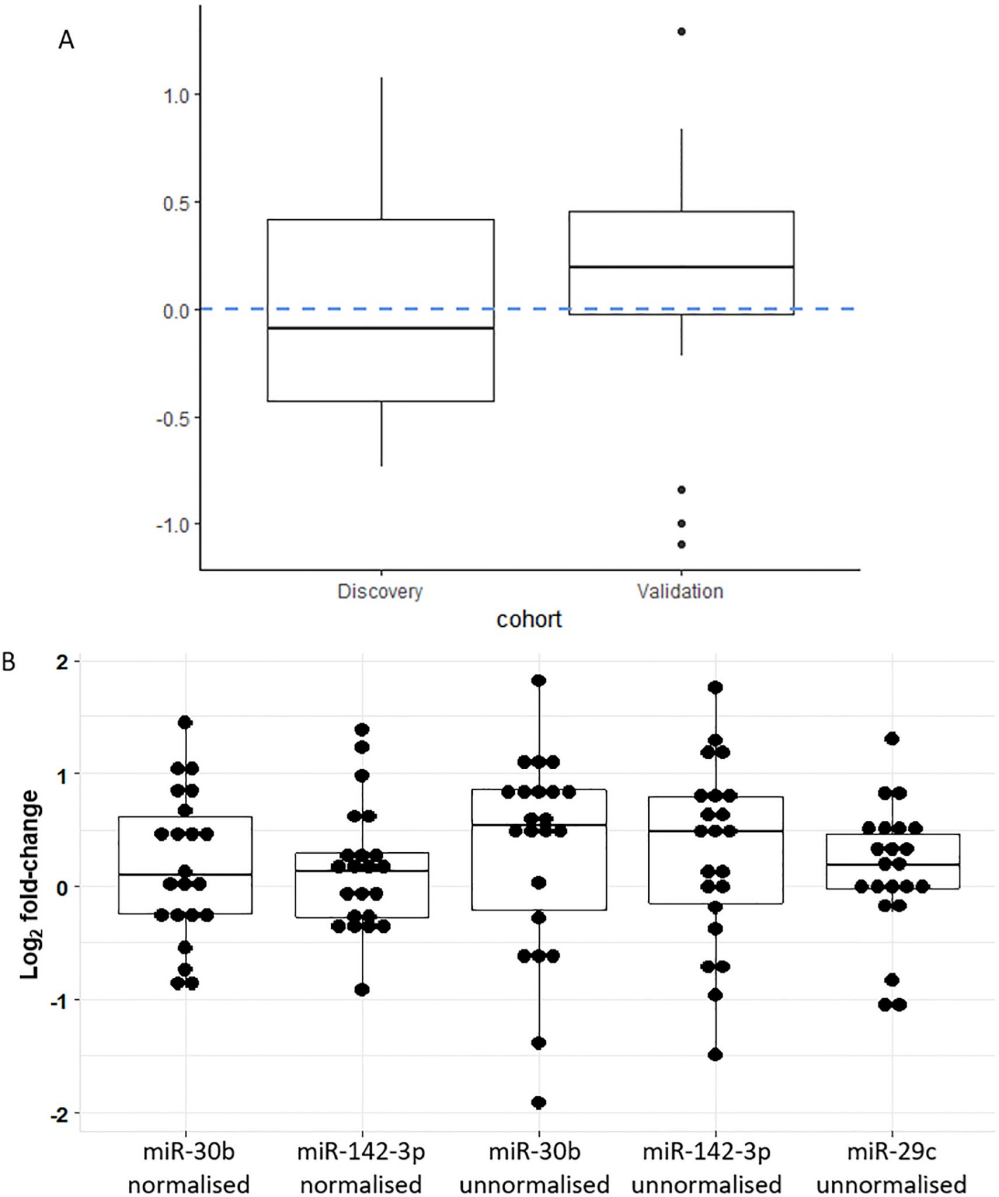
Lack of validation of differential expression of miR-142-3p is not due to the performance of miR-29c as a reference gene. (A) Log_2_ fold-change in the expression of miR-29c in the pilot and validation cohorts. This plot suggests that miR-29c did not perform better in the pilot cohort compared with the validation cohort as the interquartile range was smaller in the validation cohort. The median expression of mR-29c was positive, which if anything, will potentially bias hsa-miR-30b and hsa-miR-142-3p towards downregulation, making validation more likely. (B) Mean log_2_ fold-change pre versus post vaccination in the validation cohort. Box plots delineates range, median, interquartile range. A log2fold-change of 0 equates no change pre versus post vaccination (i.e. a fold-change of 1). Had miR-29c been downregulated after vaccination in the validation cohort (and therefore been an unsuitable normaliser) the this would have artificially elevated log2fold-changes for miR-142-3p but there is no evidence for this. The graph shows miR-29c was more stably expressed pre versus post vaccination than miR-30b and miR-142-3p, supporting its use a normaliser. Normalised fold-changes for miR-30b and miR-142-3p were lower and less variable compared with unnormalised fold-changes—this is in keeping with the removal of technical noise through use of 29c as normaliser.

Fig 8 shows that neither of these scenarios were likely—miR-29c expression was not more variable in the validation cohort compared with the discovery cohort, and non-normalised log_2_ fold-changes do not suggest that miR-29c is unlikely to be downregulated post vaccination. Non-normalised log2 fold-changes in miR-142-3p expression, were generally positive. After normalisation with miR-29c, pre versus post vaccine miR-142-3p log_2_ fold-changes were generally less positive and less variable. This suggests that lack of differential expression was not due to poor performance of miR-29c as a normaliser in the validation cohort.

## Discussion

This was the first study to use global profiling techniques to investigate miRNA expression in serum post vaccination. Agilent array analysis identified differential expression of 19 miRNAs after correction for multiple testing. This finding supports work by others showing vaccination alters serum miRNA expression[19,46]. For example, Xiong et al found that increased serum miRNA-155 at 4 to 6 weeks post hepatitis B vaccination was associated with non-response to the vaccine (defined as anti-HBsAg antibody levels below 10 mIU/ml)[46]. De Candia et al. found that there were elevated levels of miR-150 in adults and children one month after flu vaccination and that this increase correlated with haemaglutinin antibody titres[19]. *In-vitro* work supported these findings, with CD4+ and B lymphocytes secreting miR-150 into cell culture medium upon mitogenic activation.

Surprisingly, four viral miRNAs expressed by: human cytomegalovirus, herpes simplex 1 virus, herpes simplex 2, and Kaposi’s sarcoma virus were significantly upregulated after vaccination. Multiple studies have identified circulating viral miRNAs in asymptomatic patients, found them to be differential expressed between clinical states[47][48]. It is tempting to speculate that activation of the immune system by pathogens or vaccine antigens could alter the ability of the immune to control latent infections leading to viral reactivation. Unfortunately, we were unable to optimise the RT-qPCR assays to validate upregulation of these four viral miRNAs, and for reasons further discussed below, these viral miRNAs may have been falsely detected by the microarray. Although it is theoretically possible that contamination is the cause of viral miRNAs being detected on the array, we believe that this is highly unlikely in practice because: 1) miRNAs from herpes simplex I, herpes simplex II and Kaposi’s sarcoma virus were detected in 100% of samples, and miRNAs from Epstein–Barr virus and human cytomegalovirus were detected in 90% of samples. Such ubiquitous contamination of samples with RNA from all these viruses is unlikely as we do not work with these viruses in our lab; 2) contamination with viruses being the cause of differential expression of viral miRNAs would require systematic addition of miRNAs from each virus to either pre or post vaccination samples (for up- and downregulated viral miRNAs respectively), which we believe unlikely, given one would expect contamination to happen in a random or universal manner; 3) if contamination with viruses was the cause for differential expression of viral miRNAs, one would expect that all the miRNAs of that virus (detected on the array) would be upregulated/downregulated, yet this is not the case as only specific miRNAs are differentially expressed for each virus; 4) we were unable to detect viral miRNAs by RTPCR.

Only 3 (miR-30b, miR-142-3p, and 483-5p) out of 19 of the differentially expressed miRNAs, and two candidate endogenous control miRNAs (miR-29c and miR-197) could be detected by RT-qPCR. Some assays showed no detectable amplification (miR-575, miR-1202) or were at the limit of detection (hcmv-miR-UL70-3p, miR-638, miR-1207), the remaining assays had significant amounts of background above which miRNAs could not be detected (see supplementary S5 Table). Difficulties in validating results across multiple platforms is not unique to this study. A study by Mestadagh et al showed average concordance between any two platforms (microarray, next generation sequencing, RT-qPCR arrays) was 79.2% in terms of detecting the presence or absence of a miRNA and only 54.6% in terms of detecting differential expression[49]. Absence of detection of several miRNAs in the present study by RT-qPCR could be because those miRNAs were never expressed in the first place or because of PCR limitations—e.g. primers not being able to amplify the desired product, or primers/PCR set up were too insensitive to detect the miRNAs because of their low abundance in serum (a previous paper notes detection of miR-575 in placentae by RT-qPCR for example [50]).

Rather than PCR being an issue, the microarrays may be the issue. Several lines of circumstantial evidence suggest that the microarrays were falsely detecting some of the differentially expressed miRNAs; a) miRNAs which are unlikely to be present in children’s serum, e.g. herpes simplex 2 miRNAs, were detected in all serum samples (S2 Fig), b) only 5 of the differentially expressed miRNAs are labelled as “high confidence of existence” in miRbase, c) many of the differentially expressed miRNAs are absent in the fantom5 sequencing database–a human miRNA expression atlas which contains the miRNA expression profile of 121 different cell types, and d) two different primer technologies failed to detect 3 out of 6 differentially expressed miRNAs and candidate reference genes (S6 Table) [5,51,52]. The florescence signals of the probes for the miRNAs that could not be detected by RT-qPCR were too high to suggest that they represented background fluorescence (S3 Fig). More likely, is that their signal has arisen through cross-hybridization with other nucleic acids. Given miRNAs are around 22 nucleotides, median probe length on the Agilent miRNA microarray used in this study in this study (16-mer, IQR: 14-mer, 18-mer) is relatively short compared with mRNA microarrays and thus could contribute to lack of specificity. The hairpin-based nature of the probes on the miRNA microarray used in this study does attempt to reduce issues of non-specific binding but may not be sufficient, especially for biofluid samples where miRNA content is close to the limit of detection. Only one microarray platform was used in this study thus the generalisation of our finding to other miRNA array-based technologies is debateable, nevertheless we would encourage future oligonucleotide hybridisation-based microarray studies, regardless of the microarray technology, to interpret their results with caution in the absence of a second validation method.

RT-qPCR could confirm differential expression in one out of the three miRNAs that could be detected. This suggests that microarrays can provide some reliable data. Despite this, the significant downregulation of miR-142-3p could not be replicated in an independent validation cohort. A challenge for validating differential expression in the independent cohort is that it is dependent on miR-29c being an effective reference gene in the validation cohort, but as Fig 8 shows, the expression of miR-29c in the validation cohort does not appear to be the reason for the lack of validation.

Lack of validation does not appear to be due to inadequate study power because: 1) the validation cohort was over 90% powered to detect significant downregulation of miR-142-3p at the same fold-change as seen in the discovery cohort and 2) average fold-change was greater than 1 for miR-142-3p post vaccination, which is the opposite direction to the discovery cohort where fold-change was less than 1.

Differences in the findings of the discovery and validation cohort is unlikely to be due to differences in the demographics of the two cohorts as there was no obvious interaction between miR-30b and miR-142-3p expression and age, sex or vaccine administered, and the population characteristics of the two cohorts were similar (S3 and S4 Tables). It is possible that despite trying to control for a type 1 error rate by using an FDR adjusted p-value, chance alone identified differential expression in the discovery cohort. Another possibility is the “winners curse” phenomenon which has been described in genetic association studies, and can lead to an overestimated effect size in the amongst differentially expressed genes, meaning that to achieve enough power, a subsequent validation cohort must be larger than the size which is calculated by a standard power calculation[53]. This study reiterates the importance of confirming results in a second independent cohort.

Finally, we note that investigating miRNA expression 3 weeks post 2^nd^ vaccination may only identify circulating miRNA changes associated with memory responses. We were limited to this timepoint however as this was the only post vaccination timepoint when serum was collected in the trial. An earlier timepoint may have captured changes in the expression of miRNAs related to the early immune response, and this would be interesting to look at in future studies.

## Conclusion

In conclusion, this is the first study to profile serum miRNA expression post vaccination. This study contributes to the miRNAome expression profiling data in healthy children which may be useful to others trying to identify appropriate endogenous reference miRNAs. The microarray data supports the concept that circulating miRNA expression is affected by vaccination but corroboration of these results by RT-qPCR was not shown for many miRNAs. When RT-qPCR could be optimised, the microarray findings were corroborated for 1 out of 3 miRNAs using RNA samples from the same cohort but these findings could not be replicated in an independent cohort. This study therefore underlines the need for a rigorous approach to the analysis and interpretation of probe-based miRNA microarrays that, as highlight by this study, should include cross platform validation and replication in a validation cohort. Based on our study we conclude that findings from studies in which this has not been done should be interpreted with caution. Alternative methods such as next generation sequencing or RT-qPCR arrays may provide a more robust way of investigating whether vaccination affects miRNA.

MiRNAs are important mediators of immunity, thus investigating associations between circulating miRNAs is a worthwhile pursuit which could bring biological insights and identify new surrogates of protection, but as this study shows, great care should be taken when interpreting omics biomarker discovery to ensure robust, reproducible conclusions.

## Supporting information

S1 Table Details of tested assays. Company assay IDs are displayed in columns 2 and 3. Assays that met criteria for acceptable amplification of target product are highlighted in bold.
(DOCX)

S2 Table Discovery cohort demographics. (DOCX)

S3 Table Validation cohort demographic. (DOCX)

S4 Table Discovery cohort broken down into vaccine and gender. Proportion of total cohort given in brackets.
(DOCX)

S5 Table Discovery cohort broken down into vaccine and gender. Proportion of total cohort given in brackets.
(DOCX)

S6 Table MiRNAs which were differentially expressed after vaccination, and evidence of their existence. Of the 15 differentially expressed *Homo sapien* miRNAs, only 4 are convincingly expressed in the Fantom 5 database. Ten out 12 of the differentially expressed miRNAs could not be detected by LNA primers when tested. Three of the differentially expressed mRNAs were assayed for using two primer technologies, of which only one could be detected.
(DOCX)

S1 Fig Principal component analysis based on each sample’s microRNA expression without correcting for participant ID. All miRNAs passing QC were included in the principle component analysis. PCA 1 and PCA 2 are shown. Pre-vaccine samples are coloured blue, post vaccine samples are coloured red. There is some overlap between the pre- and post- vaccine sample clusters, nevertheless, there is a tendency for pre-vaccine sample to cluster to lower left, and pre-vaccine samples to cluster to upper right. This suggests that some but not all variation in global microRNA expression is accounted for by vaccine status. The plot shows that even without adjustment for pairing in the data, pre and post vaccine samples cluster somewhat separately.
(TIF)

S2 Fig Box plots of normalised signal intensity of miRNAs in each sample. Expression of hsv2 miRNAs have been plotted in blue. Black dots are outliers in the boxplot. The plot shows that hsv2 miRNAs were ubiquitously detected across all samples. This is biologically unlikely given the low prevalence of hsv2 infection in young children.
(TIF)

S3 Fig Box plots of normalised signal intensity of miRNAs in each sample. Expression of the differentially expressed miRNAs and candidate reference miRNAs have been over plotted. The plot shows that the majority of miRNAs that were differentially expressed/selected as candidate endogenous reference miRNAs were relatively well expressed compared with the lower limit of detection.
(TIF)

S1 Results Sample size estimate for validation cohort based on estimates derived from the discovery cohort. (DOCX)

S1 Dataset RT-qPCR data for discovery and validation cohorts. (XLSX)

## References

[1] Li S, Rouphael N, Duraisingham S, Romero-Steiner S, Presnell S, Davis C, et al Molecular signatures of antibody responses derived from a systems biology study of five human vaccines. Nat Immunol. 2013;15: 195–204. 10.1038/ni.2789 24336226 PMC3946932

[2] O’Connor D, Pollard AJ. Characterizing vaccine responses using host genomic and transcriptomic analysis. Clin Infect Dis. 2013;57: 860–9. 10.1093/cid/cit373 23728145

[3] Letvin NL, Rao SS, Montefiori DC, Seaman MS, Sun Y, Lim S-Y, et al Immune and Genetic Correlates of Vaccine Protection Against Mucosal Infection by SIV in Monkeys. Sci Transl Med. 2011;3: 81ra36–81ra36. 10.1126/scitranslmed.3002351 21543722 PMC3718279

[4] Jonas S, Izaurralde E. Towards a molecular understanding of microRNA-mediated gene silencing. Nat Rev Genet. 2015;16: 421–33. 10.1038/nrg3965 26077373

[5] MiRbase version 22 [Internet]. [cited 18 Jan 2017]. http://www.mirbase.org/

[6] Friedman RC, Farh KK-H, Burge CB, Bartel DP. Most mammalian mRNAs are conserved targets of microRNAs. Genome Res. Cold Spring Harbor Laboratory Press; 2009;19: 92–105. 10.1101/gr.082701.108 18955434 PMC2612969

[7] Duval M, Cossart P, Lebreton A. Mammalian microRNAs and long noncoding RNAs in the host-bacterial pathogen crosstalk. Semin Cell Dev Biol. 2016; 10.1016/j.semcdb.2016.06.016 27381344 PMC7089780

[8] Foster PS, Plank M, Collison A, Tay HL, Kaiko GE, Li J, et al The emerging role of microRNAs in regulating immune and inflammatory responses in the lung. Immunol Rev. 2013;253: 198–215. 10.1111/imr.12058 23550648

[9] Chen C-Z, Li L, Lodish HF, Bartel DP. MicroRNAs Modulate Hematopoietic Lineage Differentiation. Science (80-). 2004;303 10.1126/science.1091903 14657504

[10] Koralov SB, Muljo SA, Galler GR, Krek A, Chakraborty T, Kanellopoulou C, et al Dicer Ablation Affects Antibody Diversity and Cell Survival in the B Lymphocyte Lineage. Cell. 2008;132: 860–874. 10.1016/j.cell.2008.02.020 18329371

[11] Bazzoni F, Rossato M, Fabbri M, Gaudiosi D, Mirolo M, Mori L, et al Induction and regulatory function of miR-9 in human monocytes and neutrophils exposed to proinflammatory signals. Proc Natl Acad Sci. 2009;106: 5282–5287. 10.1073/pnas.0810909106 19289835 PMC2664036

[12] Smyth LA, Boardman DA, Tung SL, Lechler R, Lombardi G. MicroRNAs affect dendritic cell function and phenotype. Immunology. 2015;144: 197–205. 10.1111/imm.12390 25244106 PMC4298414

[13] Steiner DF, Thomas MF, Hu JK, Yang Z, Babiarz JE, Allen CDC, et al MicroRNA-29 Regulates T-Box Transcription Factors and Interferon-γ Production in Helper T Cells. Immunity. 2011;35: 169–181. 10.1016/j.immuni.2011.07.009 21820330 PMC3361370

[14] Rossato M, Curtale G, Tamassia N, Castellucci M, Mori L, Gasperini S, et al IL-10-induced microRNA-187 negatively regulates TNF-α, IL-6, and IL-12p40 production in TLR4-stimulated monocytes. Proc Natl Acad Sci U S A. 2012;109: E3101–10. 10.1073/pnas.1209100109 23071313 PMC3494907

[15] Lecellier C-H, Dunoyer P, Arar K, Lehmann-Che J, Eyquem S, Himber C, et al A cellular microRNA mediates antiviral defense in human cells. Science. 2005;308: 557–60. 10.1126/science.1108784 15845854

[16] Bai XT, Nicot C. miR-28-3p is a cellular restriction factor that inhibits human T cell leukemia virus, type 1 (HTLV-1) replication and virus infection. J Biol Chem. 2015;290: 5381–90. 10.1074/jbc.M114.626325 25568327 PMC4342455

[17] He J, Ji Y, Li A, Zhang Q, Song W, Li Y, et al MiR-122 directly inhibits human papillomavirus E6 gene and enhances interferon signaling through blocking suppressor of cytokine signaling 1 in SiHa cells. PLoS One. 2014;9: e108410 10.1371/journal.pone.0108410 25265013 PMC4180754

[18] Zheng Z, Ke X, Wang M, He S, Li Q, Zheng C, et al Human microRNA hsa-miR-296-5p suppresses enterovirus 71 replication by targeting the viral genome. J Virol. 2013;87: 5645–56. 10.1128/JVI.02655-12 23468506 PMC3648165

[19] de Candia P, Torri A, Gorletta T, Fedeli M, Bulgheroni E, Cheroni C, et al Intracellular modulation, extracellular disposal and serum increase of MiR-150 mark lymphocyte activation. PLoS One. 2013;8: e75348 10.1371/journal.pone.0075348 24205408 PMC3805464

[20] Vickers KC, Palmisano BT, Shoucri BM, Shamburek RD, Remaley AT. MicroRNAs are transported in plasma and delivered to recipient cells by high-density lipoproteins. Nat Cell Biol. Nature Research; 2011;13: 423–433. 10.1038/ncb2210 21423178 PMC3074610

[21] Arroyo JD, Chevillet JR, Kroh EM, Ruf IK, Pritchard CC, Gibson DF, et al Argonaute2 complexes carry a population of circulating microRNAs independent of vesicles in human plasma. Proc Natl Acad Sci. National Academy of Sciences; 2011;108: 5003–5008. 10.1073/pnas.1019055108 21383194 PMC3064324

[22] Zernecke A, Bidzhekov K, Noels H, Shagdarsuren E, Gan L, Denecke B, et al Delivery of microRNA-126 by apoptotic bodies induces CXCL12-dependent vascular protection. Sci Signal. 2009;2: ra81. 10.1126/scisignal.2000610 19996457

[23] Irmak MK, Erdem U, Kubar A. Antiviral activity of salivary microRNAs for ophthalmic herpes zoster. Theor Biol Med Model. 2012;9: 21 10.1186/1742-4682-9-21 22676898 PMC3422169

[24] Weber JA, Baxter DH, Zhang S, Huang DY, Huang KH, Lee MJ, et al The microRNA spectrum in 12 body fluids. Clin Chem. 2010;56: 1733–41. 10.1373/clinchem.2010.147405 20847327 PMC4846276

[25] Turchinovich A, Tonevitsky AG, Burwinkel B. Extracellular miRNA: A Collision of Two Paradigms. Trends Biochem Sci. 2016;41: 883–892. 10.1016/j.tibs.2016.08.004 27597517

[26] Drury RE, O’Connor D, Pollard AJ. The Clinical Application of MicroRNAs in Infectious Disease. Front Immunol. 2017;8: 1182 10.3389/fimmu.2017.01182 28993774 PMC5622146

[27] Nakaya HI, Hagan T, Duraisingham SS, Lee EK, Kwissa M, Rouphael N, et al Systems Analysis of Immunity to Influenza Vaccination across Multiple Years and in Diverse Populations Reveals Shared Molecular Signatures. Immunity. 2015;43: 1186–98. 10.1016/j.immuni.2015.11.012 26682988 PMC4859820

[28] Chen X, Ba Y, Ma L, Cai X, Yin Y, Wang K, et al Characterization of microRNAs in serum: a novel class of biomarkers for diagnosis of cancer and other diseases. Cell Res. 2008;18: 997–1006. 10.1038/cr.2008.282 18766170

[29] Haralambieva IH, Kennedy RB, Simon WL, Goergen KM, Grill DE, Ovsyannikova IG, et al Differential miRNA expression in B cells is associated with inter-individual differences in humoral immune response to measles vaccination. Roemer K, editor. PLoS One. Public Library of Science; 2018;13: e0191812 10.1371/journal.pone.0191812 29381765 PMC5790242

[30] Fukuyama Y, Yuki Y, Katakai Y, Harada N, Takahashi H, Takeda S, et al Nanogel-based pneumococcal surface protein A nasal vaccine induces microRNA-associated Th17 cell responses with neutralizing antibodies against Streptococcus pneumoniae in macaques. Mucosal Immunol. 2015;8: 1144–53. 10.1038/mi.2015.5 25669148 PMC4762909

[31] Waddington CS, Walker WT, Oeser C, Reiner A, John T, Wilkins S, et al Safety and immunogenicity of AS03B adjuvanted split virion versus non-adjuvanted whole virion H1N1 influenza vaccine in UK children aged 6 months-12 years: open label, randomised, parallel group, multicentre study. BMJ. 2010;340: c2649 10.1136/bmj.c2649 20508026 PMC2877808

[32] Ellis JS, Zambon MC. Molecular analysis of an outbreak of influenza in the United Kingdom. Eur J Epidemiol. Kluwer Academic Publishers; 1997;13: 369–372. 10.1023/A:1007391222905 PMC7088088 9258541

[33] Rowe T, Abernathy RA, Hu-Primmer J, Thompson WW, Lu X, Lim W, et al Detection of antibody to avian influenza A (H5N1) virus in human serum by using a combination of serologic assays. J Clin Microbiol. American Society for Microbiology (ASM); 1999;37: 937–43. 10.1128/jcm.37.4.937-943.1999 PMC88628 10074505

[34] Agilent. Agilent miRNA protocol (Version 2.4 September 2011).

[35] R Core Team (2017). R: A Language and Environment for Statistical Computingle. R Found Stat Comput Vienna, Austria. 2017;

[36] Huber W, von Heydebreck A, Sultmann H, Poustka A, Vingron M. Variance stabilization applied to microarray data calibration and to the quantification of differential expression. Bioinformatics. Narnia; 2002;18: S96–S104. 10.1093/bioinformatics/18.suppl_1.S96 12169536

[37] Lawlor N, Fabbri A, Guan P, George J, Karuturi RKM. multiClust: An R-package for Identifying Biologically Relevant Clusters in Cancer Transcriptome Profiles. Cancer Inform. Libertas Academica; 2016;15: 103–14. 10.4137/CIN.S38000 27330269 PMC4907340

[38] Kirschner MB, Kao SC, Edelman JJ, Armstrong NJ, Vallely MP, van Zandwijk N, et al Haemolysis during Sample Preparation Alters microRNA Content of Plasma. Pfeffer S, editor. Public Library of Science; 2011;6: e24145 10.1371/journal.pone.0024145 21909417 PMC3164711

[39] Benz F, Roderburg C, Vargas Cardenas D, Vucur M, Gautheron J, Koch A, et al U6 is unsuitable for normalization of serum miRNA levels in patients with sepsis or liver fibrosis. Exp Mol Med. Korean Society for Biochemistry and Molecular Biology; 2013;45: e42 10.1038/emm.2013.81 24052167 PMC3789266

[40] Kirschner MB, Kao SC, Edelman JJ, Armstrong NJ, Vallely MP, van Zandwijk N, et al No Title. Pfeffer S, editor. Public Library of Science; 2011;6: e24145. 10.1371/journal.pone.0024145 PMC3164711 21909417

[41] Moldovan L, Batte KE, Trgovcich J, Wisler J, Marsh CB, Piper M. Methodological challenges in utilizing miRNAs as circulating biomarkers. J Cell Mol Med. Wiley-Blackwell; 2014;18: 371–90. 10.1111/jcmm.12236 24533657 PMC3943687

[42] Jensen SG, Lamy P, Rasmussen MH, Ostenfeld MS, Dyrskjøt L, Orntoft TF, et al Evaluation of two commercial global miRNA expression profiling platforms for detection of less abundant miRNAs. BMC Genomics. BioMed Central; 2011;12: 435 10.1186/1471-2164-12-435 21867561 PMC3184117

[43] Ballantyne KN, van Oorschot RAH, Mitchell RJ. Locked nucleic acids in PCR primers increase sensitivity and performance. Genomics. Academic Press; 2008;91: 301–305. 10.1016/J.YGENO.2007.10.016 18164179

[44] Braasch DA, Corey DR. Locked nucleic acid (LNA): fine-tuning the recognition of DNA and RNA. Chem Biol. Elsevier; 2001;8: 1–7. 10.1016/S1074-5521(00)00058-2 11182314

[45] Mestdagh P, Hartmann N, Baeriswyl L, Andreasen D, Bernard N, Chen C, et al Evaluation of quantitative miRNA expression platforms in the microRNA quality control (miRQC) study. Nat Methods. 2014;11: 809–815. 10.1038/nmeth.3014 24973947

[46] Xiong Y, Chen S, Liu L, Zhao Y, Lin W, Ni J. Increased serum microRNA-155 level associated with nonresponsiveness to hepatitis B vaccine. Clin Vaccine Immunol. American Society for Microbiology (ASM); 2013;20: 1089–91. 10.1128/CVI.00044-13 23637039 PMC3697454

[47] Li S, Zhu J, Zhang W, Chen Y, Zhang K, Popescu LM, et al Signature microRNA expression profile of essential hypertension and its novel link to human cytomegalovirus infection. Circulation. 2011;124: 175–84. 10.1161/CIRCULATIONAHA.110.012237 21690488

[48] Tudor S, Giza DE, Lin HY, Fabris L, Yoshiaki K, D’Abundo L, et al Cellular and Kaposi’s sarcoma-associated herpes virus microRNAs in sepsis and surgical trauma. Cell Death Dis. 2014;5: e1559 10.1038/cddis.2014.515 25476907 PMC4649832

[49] Mestdagh P, Hartmann N, Baeriswyl L, Andreasen D, Bernard N, Chen C, et al Evaluation of quantitative miRNA expression platforms in the microRNA quality control (miRQC) study. Nat Methods. 2014;11: 809–15. 10.1038/nmeth.3014 24973947

[50] Hiura H, Hattori H, Kobayashi N, Okae H, Chiba H, Miyauchi N, et al Genome-wide microRNA expression profiling in placentae from frozen-thawed blastocyst transfer. Clin Epigenetics. 2017;9: 79 10.1186/s13148-017-0379-6 28785370 PMC5543431

[51] Vyse AJ, Gay NJ, Slomka MJ, Gopal R, Gibbs T, Morgan-Capner P, et al The burden of infection with HSV-1 and HSV-2 in England and Wales: implications for the changing epidemiology of genital herpes. Sex Transm Infect. BMJ Group; 2000;76: 183–7. 10.1136/sti.76.3.183 10961195 PMC1744133

[52] de Rie D, Abugessaisa I, Alam T, Arner E, Arner P, Ashoor H, et al An integrated expression atlas of miRNAs and their promoters in human and mouse. Nature Biotechnology. Nature Publishing Group; 2017 pp. 872–878. 10.1038/nbt.3947 28829439 PMC5767576

[53] Xiao R, Boehnke M. Quantifying and correcting for the winner’s curse in genetic association studies. Genet Epidemiol. NIH Public Access; 2009;33: 453–62. 10.1002/gepi.20398 19140131 PMC2706290

