# Supplementary material for: The effect of H1N1 vaccination on serum miRNA expression in children: A tale of caution for microRNA microarray studies
Source: PLoS One. 2019 Aug 20;14(8):e0221143. doi: 10.1371/journal.pone.0221143 (PMC6701777; doi:10.1371/journal.pone.0221143)
Supplement: S1 Table — Company assay IDs are displayed in columns 2 and 3. Assays that met criteria for acceptable amplification of target product are highlighted in bold. (DOCX) [file pone.0221143.s001.docx]

| Reason for testing | MiRNA | miRNA Locked Nucleic Acid (LNA™) primer sets (Exiqon)  Cat # 339306 | TaqMan™ advanced miRNA assays  Cat # A25576 |
| --- | --- | --- | --- |
| Differentially expressed in the microarray miRNA data | hsa-miR-575 | 2114484 | 479056_mir |
|  | hsa-miR-4270 | 2114586 | - |
|  | hsa-miR-483-5p | 205693 | **478432_mir** |
|  | hsa-miR-3679-5p | 2109537 | - |
|  | hsa-miR-1207-5p | 204693 | - |
|  | hsa-miR-1202 | 206001 | - |
|  | hcmv-miR-U70-3p | 205856 | - |
|  | hsa-miR-638 | 204234 | 478187_mir |
|  | hsv2-miR-H25 | 206029 | - |
|  | hsv1-miR-H17 | 205759 | - |
|  | hsa-miR-30b | **204765** | - |
|  | hsa-miR-142-3 | **204291** | - |
|  | Hsa-miR-671-5p | 205649 | - |
| Candidate reference miRNAs | hsa-miR-4281 | 2100243 |  |
|  | hsa-miR-29c-3p | **204729** | **479229_mir** |
|  | hsa-miR-3665 | 2100158 | 479701_mir |
|  | hsa-miR-3162- 5p | 2107673 | - |
|  | hsa-miR-1249 | 204122 | - |
|  | hsa-miR-197-3p | 204380 | - |

TABLE S1: Details of tested assays.

Assays that met the criteria for acceptable amplification are highlighted in bold
