# Supplementary material for: The effect of H1N1 vaccination on serum miRNA expression in children: A tale of caution for microRNA microarray studies
Source: PLoS One. 2019 Aug 20;14(8):e0221143. doi: 10.1371/journal.pone.0221143 (PMC6701777; doi:10.1371/journal.pone.0221143)
Supplement: S2 Table — (DOCX) [file pone.0221143.s002.docx]

**TABLE S2. Discovery Cohort demographics**

| Vaccine Type | Gender | Age (years) |
| --- | --- | --- |
| AS03B adjuvanted split virion vaccine | F | 4.9 |
| AS03B adjuvanted split virion vaccine | F | 7.5 |
| AS03B adjuvanted split virion vaccine | F | 10.4 |
| AS03B adjuvanted split virion vaccine | F | 1.0 |
| AS03B adjuvanted split virion vaccine | F | 5.6 |
| AS03B adjuvanted split virion vaccine | F | 4.3 |
| Non-adjuvanted whole virion vaccine | F | 8.9 |
| Non-adjuvanted whole virion vaccine | F | 11.8 |
| Non-adjuvanted whole virion vaccine | F | 1.3 |
| Non-adjuvanted whole virion vaccine | F | 6.0 |
| Non-adjuvanted whole virion vaccine | F | 1.8 |
| Non-adjuvanted whole virion vaccine | F | 1.3 |
| AS03B adjuvanted split virion vaccine | M | 3.8 |
| AS03B adjuvanted split virion vaccine | M | 9.6 |
| AS03B adjuvanted split virion vaccine | M | 1.5 |
| Non-adjuvanted whole virion vaccine | M | 9.1 |
| Non-adjuvanted whole virion vaccine | M | 3.2 |
| Non-adjuvanted whole virion vaccine | M | 9.4 |
| Non-adjuvanted whole virion vaccine | M | 5.8 |
| Non-adjuvanted whole virion vaccine | M | 3.4 |
| Non-adjuvanted whole virion vaccine | M | 12.1 |
| Non-adjuvanted whole virion vaccine | M | 10.4 |
