# Supplementary material for: The effect of H1N1 vaccination on serum miRNA expression in children: A tale of caution for microRNA microarray studies
Source: PLoS One. 2019 Aug 20;14(8):e0221143. doi: 10.1371/journal.pone.0221143 (PMC6701777; doi:10.1371/journal.pone.0221143)
Supplement: S3 Table — (DOCX) [file pone.0221143.s003.docx]

| Vaccine Type | gender | Age (years) |
| --- | --- | --- |
| AS03B adjuvanted split virion vaccine | f | 3.0 |
| AS03B adjuvanted split virion vaccine | f | 1.9 |
| AS03B adjuvanted split virion vaccine | f | 1.9 |
| AS03B adjuvanted split virion vaccine | f | 5.9 |
| Non-adjuvanted whole virion vaccine | f | 2.5 |
| Non-adjuvanted whole virion vaccine | f | 9.7 |
| Non-adjuvanted whole virion vaccine | f | 12.3 |
| AS03B adjuvanted split virion vaccine | m | 2.4 |
| AS03B adjuvanted split virion vaccine | m | 2.9 |
| AS03B adjuvanted split virion vaccine | m | 6.9 |
| AS03B adjuvanted split virion vaccine | m | 4.2 |
| AS03B adjuvanted split virion vaccine | m | 6.2 |
| Non-adjuvanted whole virion vaccine | m | 12.3 |
| Non-adjuvanted whole virion vaccine | m | 2.9 |
| Non-adjuvanted whole virion vaccine | m | 2.0 |
| Non-adjuvanted whole virion vaccine | m | 1.6 |
| Non-adjuvanted whole virion vaccine | m | 2.9 |
| Non-adjuvanted whole virion vaccine | m | 3.3 |
| Non-adjuvanted whole virion vaccine | f | 11.8 |
| AS03B adjuvanted split virion vaccine | f | 10.7 |
| Non-adjuvanted whole virion vaccine | f | 10.7 |
| AS03B adjuvanted split virion vaccine | f | 4.3 |

## **TABLE S3. Validation Cohort demographics**
