# Supplementary material for: The effect of H1N1 vaccination on serum miRNA expression in children: A tale of caution for microRNA microarray studies
Source: PLoS One. 2019 Aug 20;14(8):e0221143. doi: 10.1371/journal.pone.0221143 (PMC6701777; doi:10.1371/journal.pone.0221143)
Supplement: S4 Table — Proportion of total cohort given in brackets. (DOCX) [file pone.0221143.s004.docx]

**TABLE S4. Exploratory cohort broken down into vaccine and gender.**

| Exploratory cohort | Male | Female |
| --- | --- | --- |
| AS03B adjuvanted split virion vaccine | 3 (0.14) | 6 (0.27) |
| Non-adjuvanted whole virion vaccine | 7 (0.32) | 6 (0.27) |

Proportion of total cohort given in brackets
