# Supplementary material for: The effect of H1N1 vaccination on serum miRNA expression in children: A tale of caution for microRNA microarray studies
Source: PLoS One. 2019 Aug 20;14(8):e0221143. doi: 10.1371/journal.pone.0221143 (PMC6701777; doi:10.1371/journal.pone.0221143)
Supplement: S5 Table — Proportion of total cohort given in brackets. (DOCX) [file pone.0221143.s005.docx]

| Validation cohort | Male | Female |
| --- | --- | --- |
| AS03B adjuvanted split virion vaccine | 4 (0.27) | 6 (0.27) |
| Non-adjuvanted whole virion vaccine | 5 (0.23) | 9 (0.23) |

**TABLE S5. Exploratory cohort broken down into vaccine and gender.**

Proportion of total cohort given in brackets
