# Supplementary material for: The effect of H1N1 vaccination on serum miRNA expression in children: A tale of caution for microRNA microarray studies
Source: PLoS One. 2019 Aug 20;14(8):e0221143. doi: 10.1371/journal.pone.0221143 (PMC6701777; doi:10.1371/journal.pone.0221143)
Supplement: S6 Table — Of the 15 differentially expressed Homo sapien miRNAs, only 4 are convincingly expressed in the Fantom 5 database. Ten out 12 of the differentially expressed miRNAs could not be detected by LNA primers when tested. Three of the differentially expressed mRNAs were assayed for using two primer technologies, of which only one could be detected. (DOCX) [file pone.0221143.s006.docx]

| TABLE S6: MiRNAs which were differentially expressed after vaccination, and evidence of their existence. | | | | | | |  |
| --- | --- | --- | --- | --- | --- | --- | --- |
| Direction of foldchange | **MiRNA** | **Mean fold change in the microarray data** | **Adjusted P-Value** | **Expression in Fantom5 database**[40] | **Has validated LNA primers?** | **LNA RT-PCR detection** | **Taqman RT-PCR**  **detection** |
| Up | hsa-miR-575 | 1.57 | **<0.001** | Not expressed | No | Fail | Fail |
|  | hsa-miR-4270 | 1.42 | <0.001 | Not expressed | No | Fail | - |
|  | hsa-miR-483-5p | 1.38 | 0.003 | Expressed | Yes | Fail | Pass |
|  | hsa-miR-3679-5p | 1.35 | 0.003 | Max count across all cell types = 11 | No | Fail | - |
|  | hsa-miR-1207-5p | 1.32 | <0.001 | 1 count in one cell types | No | Fail | - |
|  | hsa-miR-1202 | 1.32 | <0.001 | Not expressed | No | Fail | - |
|  | hcmv-miR-UL70-3p | 1.29 | 0.021 | NA | No | Fail | - |
|  | hsa-miR-638 | 1.28 | 0.002 | Not expressed | Yes | Fail | Fail |
|  | hsa-miR-3141 | 1.28 | 0.010 | Max count across all cell types = 4 | No | - | - |
|  | hsv2-miR-H25 | 1.28 | 0.042 | NA | Yes | Fail | - |
|  | hsa-miR-2861 | 1.27 | 0.003 | Max count across all cell types = 3 | No | - | - |
|  | hsa-miR-3196 | 1.26 | 0.010 | Max count across all cell types = 5 | No | - | - |
|  | kshv-miR-K12-3 | 1.25 | 0.003 | NA | No | - |  |
|  | hsa-miR-3656 | 1.20 | 0.014 | Max count across all cell types = 2 | No | - | - |
|  | hsv1-miR-H17 | 1.20 | 0.012 | Not expressed | Yes | Fail | - |
|  | hsa-miR-642b-3p | 1.20 | 0.049 | Max count across all cell types = 4 | No | - | - |
| Down | hsa-miR-30b | 0.80 | 0.014 | Expressed | Yes | Pass | - |
|  | hsa-miR-671-5p | 0.78 | 0.048 | Expressed | No | - | - |
|  | hsa-miR-142-3p | 0.77 | 0.047 | Expressed | Yes | Pass | - |

Of the 15 differentially expressed *Homo sapien* miRNAs, only 4 are convincingly expressed in the Fantom 5 database. Ten out 12 tested of the differentially expressed miRNAs (tested by RT-PCR) could not be detected by LNA primers. Three of the differentially expressed miRNAs were assayed for using two primer technologies, of which only one could be detected. Max count across cell types means the sum count of that miRNA across all cell types in the Fantom 5 database.
