# Supplementary figures and images for: The effect of H1N1 vaccination on serum miRNA expression in children: A tale of caution for microRNA microarray studies
Source: PLoS One. 2019 Aug 20;14(8):e0221143. doi: 10.1371/journal.pone.0221143 (PMC6701777; doi:10.1371/journal.pone.0221143)

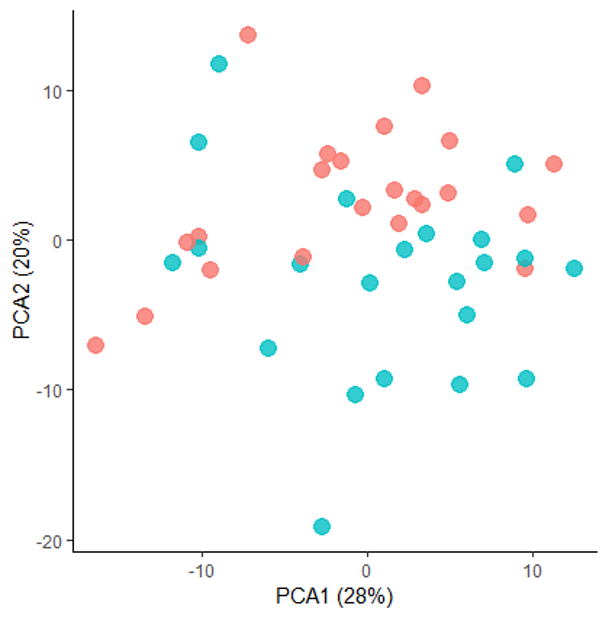

Supplement: S1 Fig — All miRNAs passing QC were included in the principle component analysis. PCA 1 and PCA 2 are shown. Pre-vaccine samples are coloured blue, post vaccine samples are coloured red. There is some overlap between the pre- and post- vaccine sample clusters, nevertheless, there is a tendency for pre-vaccine sample to cluster to lower left, and pre-vaccine samples to cluster to upper right. This suggests that some but not all variation in global microRNA expression is accounted for by vaccine status. The plot shows that even without adjustment for pairing in the data, pre and post vaccine samples cluster somewhat separately. (TIF) [file pone.0221143.s007.tif]

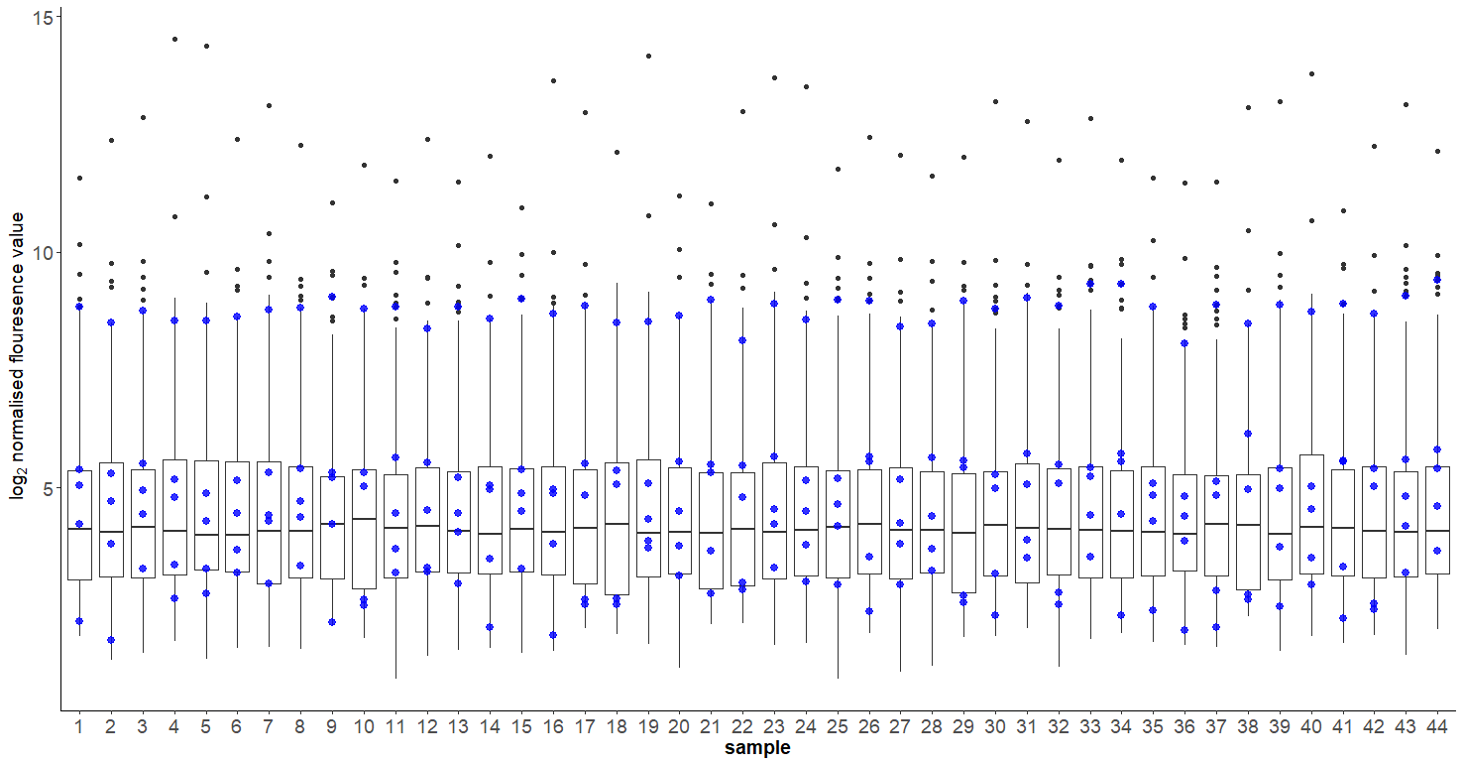

Supplement: S2 Fig — Expression of hsv2 miRNAs have been plotted in blue. Black dots are outliers in the boxplot. The plot shows that hsv2 miRNAs were ubiquitously detected across all samples. This is biologically unlikely given the low prevalence of hsv2 infection in young children. (TIF) [file pone.0221143.s008.tif]

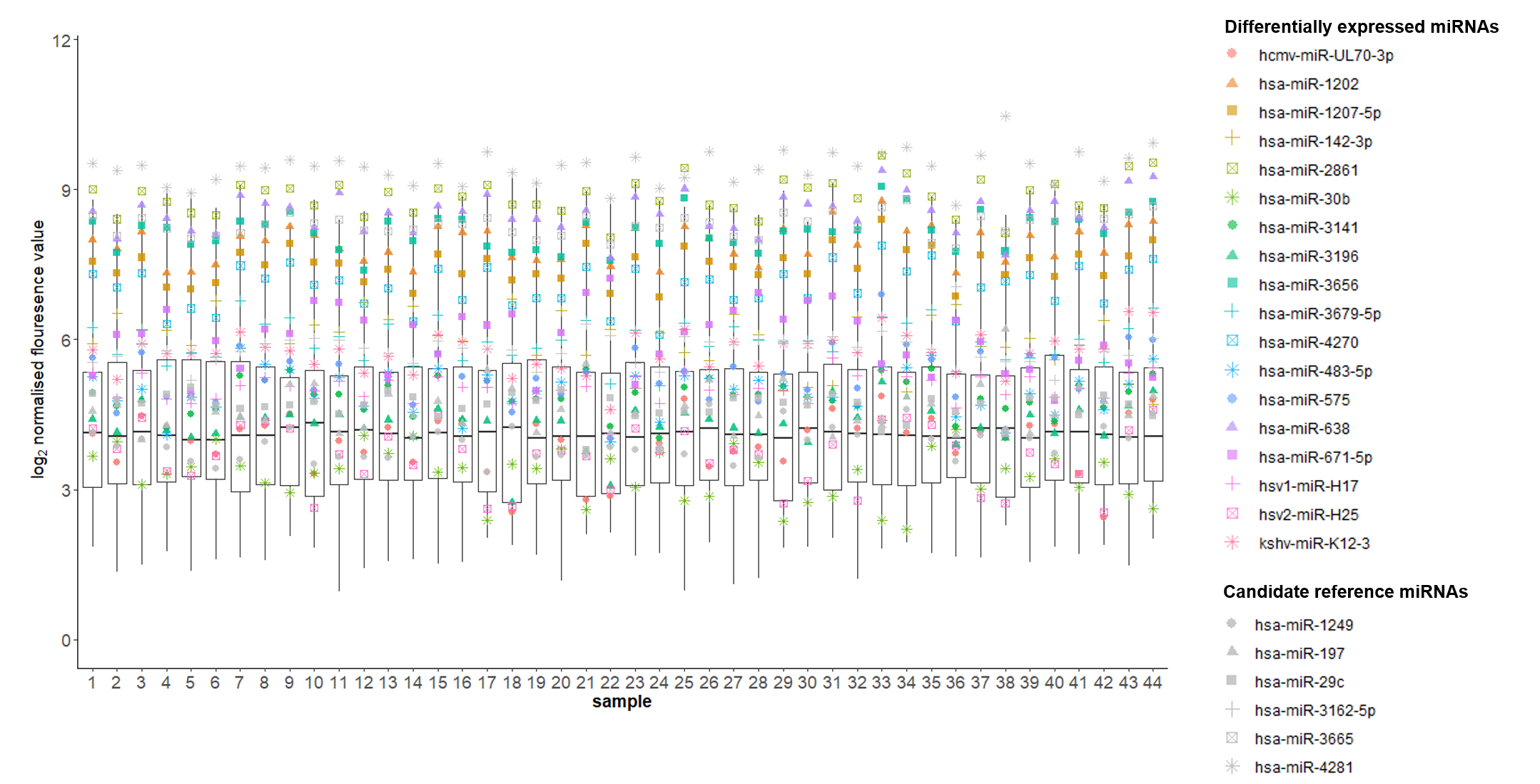

Supplement: S3 Fig — Expression of the differentially expressed miRNAs and candidate reference miRNAs have been over plotted. The plot shows that the majority of miRNAs that were differentially expressed/selected as candidate endogenous reference miRNAs were relatively well expressed compared with the lower limit of detection. (TIF) [file pone.0221143.s009.tif]
