# Supplementary material for: The effect of H1N1 vaccination on serum miRNA expression in children: A tale of caution for microRNA microarray studies
Source: PLoS One. 2019 Aug 20;14(8):e0221143. doi: 10.1371/journal.pone.0221143 (PMC6701777; doi:10.1371/journal.pone.0221143)
Supplement: S1 Results — (DOCX) [file pone.0221143.s010.docx]

**Supplementary Results:** **Sample size estimate for validation cohort based on estimates derived from the discovery cohort.**

The function pwr.t.test in the “pwr” R package was used to estimate the sample size for the validation cohort[1]. This function implements the method described by Cohen[2]. We used the following parameters to perform a power calculation for a one-sample, one-tailed t-test based on log_2_ fold-change in miR-142-3p in the discovery cohort:

| μ_0_ | μ_1_ | σ | α | β | Power | N |
| --- | --- | --- | --- | --- | --- | --- |
| 0 | -0.312 | 0.445 | 0.05 | 0.15 | 0.9 | 19 |

Where:

**μ_0_** represents the mean under the null hypothesis i.e. log_2_ fold-change after vaccination is 0

**μ_1_** represents the population mean i.e. mean log_2_ fold-change in expression of miR-142-3p as estimated from discovery cohort

**σ** represents the population standard deviation i.e. standard deviation of miR-142-3p log_2_ fold-change as estimated from discovery cohort

**α** represents the significance threshold below which the null hypothesis will be rejected i.e. p<0.05

**β** represents the type II error rate i.e. 0.15

**Power** = **1-β**

**N** = sample size i.e. number of paired samples (rounded up to nearest whole number) which fulfils the criteria specified above.

A sample size of **22 people** provides **93.8%** power to reject the null hypothesis at a significance of 0.05 given the parameters estimated from the RTPCR results in the discovery cohort. If using a two-tailed t-test 22 people provides 88% power to reject the null hypothesis.

**References**

1. Champely, Stephane. pwr: Basic Functions for Power Analysis. R Packag version 12-2. 2018;

2. Cohen J. Statistical Power Analysis for the Behavioral Sciences. Routledge; 1988. doi:10.4324/9780203771587
